# Serum N-propeptide of collagen IIA (PIIANP) as a marker of radiographic osteoarthritis burden

**DOI:** 10.1371/journal.pone.0190251

**Published:** 2017-12-29

**Authors:** Hikmat N. Daghestani, Joanne M. Jordan, Jordan B. Renner, Michael Doherty, A. Gerry Wilson, Virginia B. Kraus

**Affiliations:** 1 Duke Molecular Physiology Institute, Duke University School of Medicine, Durham, NC, United States of America; 2 Thurston Arthritis Research Center, University of North Carolina at Chapel Hill, NC, United States of America; 3 Department of Radiology, University of North Carolina at Chapel Hill, NC, United States of America; 4 Department of Academic Rheumatology, University of Nottingham, Nottingham, United Kingdom; 5 Conway Institute for Biomolecuar & Biomedical Research University College Dublin, Dublin, Ireland; 6 Division of Rheumatology, Department of Medicine, Duke University School of Medicine, Durham, NC, United States of America; Semmelweis Egyetem, HUNGARY

## Abstract

**Objective:**

Cartilage homeostasis relies on a balance of catabolism and anabolism of cartilage matrix. Our goal was to evaluate the burden of radiographic osteoarthritis and serum levels of type IIA procollagen amino terminal propeptide (sPIIANP), a biomarker representing type II collagen synthesis, in osteoarthritis.

**Methods:**

OA burden was quantified on the basis of radiographic features as total joint faces with an osteophyte, joint space narrowing, or in the spine, disc space narrowing. sPIIANP was measured in 1,235 participants from the Genetics of Generalized Osteoarthritis study using a competitive enzyme-linked immunosorbent assay. Separate multivariable linear regression models, adjusted for age, sex, and body mass index and additionally for ipsilateral osteophytes or joint/disc space narrowing, were used to assess the independent association of sPIIANP with osteophytes and with joint/disc space narrowing burden in knees, hips, hands and spine, individually and together.

**Results:**

After full adjustment, sPIIANP was significantly associated with a lesser burden of hip joint space narrowing and knee osteophytes. sPIIANP was associated with a lesser burden of hand joint space narrowing but a greater burden of hand osteophytes; these results were only evident upon adjustment for osteoarthritic features in all other joints. There were no associations of sPIIANP and features of spine osteoarthritis.

**Conclusions:**

Higher cartilage collagen synthesis, as reflected in systemic PIIANP concentrations, was associated with lesser burden of osteoarthritic features in lower extremity joints (knees and hips), even accounting for osteoarthritis burden in hands and spine, age, sex and body mass index. These results suggest that pro-anabolic agents may be appropriate for early treatment to prevent severe lower extremity large joint osteoarthritis.

## Introduction

Two major hallmarks of osteoarthritis (OA) include the irreversible loss of cartilage by degradation and new bone formation and bone remodeling[1]. Cartilage homeostasis relies on the controlled catabolism of matrix proteins such as collagen and the replacement with newly synthesized proteins by chondrocytes[2]. Type II collagen, the major protein and collagen type in articular cartilage, is synthesized as a procollagen with a propeptide domain[3]. The N-propeptide of collagen IIA (PIIANP) is a product of a specific splice form of type II collagen that is synthesized by chondroprogenitor cells[4]. PIIANP is synthesized by chondrocytes in the context of OA, possibly as an attempted repair response[5] and has been considered a marker reflecting cartilage turnover[6]. Relative to controls, low serum (s)PIIANP was shown by an ELISA method of quantification[7], to be associated with radiographic knee OA[8] and recently, knee OA progression[9]. The combination of low sPIIANP and increased urinary carboxy-terminal telopeptide of type II collagen (uCTX-II), a degradation product of type II cartilage, predict progression of radiographic knee OA in several studies[9, 10]. A recent study demonstrated that there was no association between sPIIANP and radiographic hip OA, despite association of uCTX-II with the presence and incidence of radiographic hip OA[11]. Levels of sPIIANP have also been shown to be lower in individuals with clinical hand OA compared to controls without clinical hand OA[12]. Recognizing that low systemic (serum) PIIANP is associated with clinical hand OA knee and radiographic knee OA and knee OA progression[9, 13], we hypothesized that low serum PIIANP would be associated with greater burden of radiographic OA at other joint sites. We tested this hypothesis by evaluating the association of sPIIANP and burden of radiographic OA within joint groups or sites (knee, hip, hand and spine) and within the body as a whole.

## Methods

### Participants

This study represents analyses of 1,235 individuals from a subset of the participants evaluated in the Genetics of Generalized Osteoarthritis (GOGO) study[14] (Table 1). This cohort consisted of community dwelling sibling pairs affected with radiographic hand OA (>3 joint bony enlargement) totaling 243 Caucasian men and 992 Caucasian women, age range 37–92 years (mean 65.4 ± 9.0 SD) and body mass index (BMI) range 14.9–61.7 kg/m^2^ (mean 28.8 ± 6.3 SD). Participants were excluded on the basis of a known clinical or radiographic diagnosis of other arthropathies, including a history or radiographic evidence of rheumatoid arthritis or psoriatic arthritis, or radiographic and serological evidence of hemochromatosis. Participants were treated with standard OA medications consisting primarily of non-steroidal anti-inflammatory agents and acetaminophen. Since there are no proven disease modifying agents for OA[15], we did not consider these relevant for the current cross-sectional study related to the biomarker PIIANP and structural severity of OA. Women of child-bearing age were required to have a negative serum pregnancy test before any radiographic imaging procedures. All samples were stored at -80 °C until analyzed. All study procedures were approved by the institutional review boards at Duke University, University of North Carolina, University of Nottingham, and University of Sheffield.

**Table 1:** Demographic characteristic of the 1235 participants.

Characteristic		Female (*n* = 992)	Male (*n* = 243)
Age, mean ± standard deviation [SD (range)], years		65.34 ± 8.99 (37–92)	65.77 ± 8.84 (41–87)
BMI, mean ± SD (range), kg/m^2^		28.75 ± 6.27 (16.7–56.1)	29.21 ± 5.50 (14.9–61.7)
Any Knee OA, n (%)	OST	607 (61.19)	165 (67.90)
	JSN	404 (40.72)	112 (46.09)
Any Hip OA, n (%)	OST	887 (89.42)	218 (89.71)
	JSN	229 (23.08)	76 (31.28)
Any Hand OA, n (%)	OST	959 (96.67)	236 (97.12)
	JSN	957 (96.47)	224 (92.18)
Any Spine OA, n (%)	OST	485 (48.89)	144 (59.26)
	DSN	368 (37.10)	94 (38.68)

Any OA defined as radiographic feature (osteophye = OST or joint space narrowing = JSN) score ≥1

### Radiographic imaging and scoring

Radiographs were obtained as previously described[14]. Briefly, a posteroanterior (PA) radiograph of each hand was performed with the beam centered on the third metacarpophalangeal (MCP) joint. A fixed-flexion PA knee radiograph was taken with the SynaFlexer™ positioning frame (Synarc, San Francisco, CA). With this platform, the feet were externally rotated 10°, the knees and thighs touched the vertical platform anteriorly, and the X-ray beam was tilted 10° caudally. Skyline views of both patellae were taken with the participant in the supine position, knees bent, and the beam angled from the feet toward the knees. An anteroposterior view of the pelvis was performed with the participant supine and feet internally rotated 10°. A lateral view of the lumbar spine (spanning L_1_–L_5_) was performed with the participant recumbent, with left side down. Radiographs for the hands, knees and hips were available for all participants (n = 1,235). Spine radiographs were obtained only on the participants ascertained at the US sites (n = 721).

Osteophytes (OST) and joint space narrowing (JSN) were scored using a standardized atlas for hands, hips, and knees[16]. Lumbar spine radiographs were scored for the presence and severity of vertebral osteophyte according to a standard atlas[17] and disc narrowing (DSN) was scored on a 0–3 scale. A single expert musculoskeletal radiologist (JBR) performed all radiographic assessments with high inter-rater reliability: weighted kappa 0.86 for appendicular joints[18]; weighted kappa 0.89 for spine DSN and 0.90 for spine OST[19]. All OST and JSN/DSN scores were dichotomized as 0 or 1 (for scores ≥1) and scores were summed by joint site thereby comprising an OA burden score representing the number of OA affected joint faces as previously described[20]. The “OA joint faces” metric we used proved in the past to be remarkably informative, suggesting that burden rather than severity is highly reflective of a systemic (serum or urine) concentration of a biomarker[20].To directly compare the contribution of one joint site vs another to the systemic biomarker concentration, the OA burden scores were normalized to the possible maximal scores for each joint site feature, specifically: tibiofemoral knee JSN 4, tibiofemoral knee OST 8; hip JSN 6, hip OST 8; hand JSN 30, hand OST 30; lumbar spine DSN 5, lumbar spine OST 10. Hand, knee and hip JSN data were available for 1,235 participants; hand, knee and hip OST data were available for 1,233 participants; spine DSN and OST data were available for 721 participants.

### Serum PIIANP quantification

Morning non-fasting blood was uniformly obtained for serum analyses. Serum was isolated, aliquoted, and stored within 4 hours of collection at -80°C until biomarker analyses were performed. Serum PIIANP was measured in 1,235 samples using a competitive enzyme-linked immunosorbent assay (ELISA) (EMD Millipore Corporation) according to the manufacturer’s protocol, with the exception that all samples were diluted 10-fold for analysis to minimize matrix effects (serum interfering substances) and to ensure that sPIIANP levels were measured within the range of detection. The reported limit of assay sensitivity was 17.2 ng/mL. The linear range of the assay was 32.8–2100 ng/ml. Five samples with sPIIANP levels above the highest level of detection were repeated at a 20-fold dilution and all achieved measurable values within the detection range of the assay at this dilution. The optical density of each well was measured using a microplate reader (Infinite® 200 PRO, Tecan Group Ltd., Switzerland) set to 450 nm with a wavelength correction set to 590 nm. Two quality control samples provided by the manufacturer, low and high, were measured in duplicate on each plate and were all within the expected concentration ranges of 42–114 ng/mL and 360–748 ng/mL, respectively. A best-fit standard curve was generated by non-linear regression analysis using the “One-site–Total” equation in GraphPad (Prism, GraphPad V. 5.0a, La Jolla, CA, USA). sPIIANP concentration values were interpolated from the curve fit and adjusted for dilution factor. The inter-assay CVs were 17.0%, 5.5%, and 6.3% for the low, high, and pooled serum controls, respectively. The overall inter-assay coefficient of variation was 9.6% (based on 19 plates). This compares favorably with the results for the PIIANP inter-assay CV of 12.3% in the large Foundation for NIH Osteoarthritis Biomarkers Consortium study for which the same kit was used but all analyses performed at LabCorp[9].

### Statistical analyses

Serum PIIANP concentrations were log (base 10) transformed to satisfy requirements for normality for purposes of statistical analysis. Overall, we excluded a total of two participants, one with a PIIANP concentration below the lower limit of detection (LLOD) of the assay and one with a PIIANP concentration 3 times the interquartile range above the upper quantile. Two participants did not have knee, hip, nor hand OST scores and were excluded from the analyses. Therefore, knee, hip, hand and PIIANP data used for the analyses were available from a total of 1,231 participants, with a total of 721 of these participants also having spine data. Multivariable linear regression models (JMP Pro 13, SAS, Cary, NC) were used to assess the association of sPIIANP concentrations with OST and JSN/DSN burden of knee, hip, hand and spine radiographic OA. Models included all joint sites and disease features with backward stepwise selection of variables. Models were also adjusted for age, sex, BMI, with and without the other radiographic feature from the same joint; *p*-values ≤0.05 were considered significant. The effect of radiographic feature and joint site on the all-inclusive model was ranked using the LogWorth statistic (–log10[*p*-value]) in JMP Pro.

## Results

Full sPIIANP data were available for 1,231 participants in the GOGO cohort. The demographics and numbers of participants with each of the 8 OA phenotypes (any Knee OST/JSN, any Hand OST/JSN, any Hip OST/JSN and any Spine OST/DSN) are summarized in Table 1. The concentrations of sPIIANP ranged from 695.1 to 15,489.4 ng/mL (median of 3,102.8 and interquartile range of 2,178.7 ng/mL). The range of sPIIANP concentrations for each OA phenotype are summarized in Table 2. Serum PIIANP concentrations were weakly associated with BMI (parameter estimate ß: 0.003, *P* = 0.009), but not with age or sex.

**Table 2:** Serum PIIANP concentrations of participants with and without OA phenotypes.

Phenotype		Concentration of sPIIANP (ng/mL)			
		Mean	Standard Deviation	Median	Range
**Knee**	**- JSN**	3,882.8	2,295.6	3,218.8	695.1–15,478.3
	**+ JSN**	3,652.2	2,225.8	2,932.7	1,116.3–15,489.4
	**- OST**	3,967.6	2,298.0	3,255.2	1,187.2–14,745.0
	**+ OST**	3,677.2	2,245.2	3,006.3	695.1–15,489.4
**Hip**	**- JSN**	3,917.3	2,379.8	3,205.0	695.1–15,489.4
	**+ JSN**	3,387.7	1,835.7	2,895.4	1,237.9–14,745.0
	**- OST**	3,635.3	2,012.2	3,011.2	1,187.2–11,936.6
	**+ OST**	3,804.9	2,297.7	3,119.1	695.1–15,489.4
**Hand**	**- JSN**	3,581.1	1,945.6	3,042.3	1,218.0–10,537.3
	**+ JSN**	3,796.1	2,282.7	3,119.1	695.1–15,489.4
	**- OST**	2,782.4	1,436.4	2,408.6	1,238.3–7,239.1
	**+ OST**	3,825.6	2,286.4	3,145.8	695.1–15,489.4
**Spine**	**- DSN**	3,441.2	2,089.0	2,789.2	695.1–15,489.4
	**+ DSN**	4,363.3	2,435.8	3,587.0	1,276.6–15,478.3
	**- OST**	2,782.4	1,436.4	2,408.6	1,238.3–7,239.1
	**+ OST**	4,402.0	2,509.0	3,653.2	1,276.6–15,489.4

The frequency of radiographic OA ranged from a low of 24.7% for any hip JSN to a high of 96.4% for any hand OST. With the exception of spine DSN, the results were consistent whether unadjusted or fully adjusted for age, sex, BMI and the alternative feature of the same joint (i.e. JSN/DSN adjustment of sPIIANP association with OST, or OST adjustment of sPIIANP association with JSN/DSN) (Table 3). After adjustment for age, sex, BMI, and the alternative radiographic feature of the same joint, sPIIANP concentrations were negatively associated with knee OST burden (ß -0.151, *P*<0.0001) and hip JSN burden (ß -0.166, *P*<0.0001) (Table 3). These results were unchanged upon controlling for all joint sites, features and age, sex and BMI in multivariable models. sPIIANP concentrations was negatively associated with hand JSN (ß -0.261, *P*<0.0001) and positively associated with hand OST (ß 0.249, *P*<0.0001) only after adjustment for all joint sites, features, and age, sex, and BMI in multivariable models. There was no association between sPIIANP concentrations and radiographic features of the spine.

**Table 3:** Associations of serum PIIANP with radiographic features of osteoarthritis in four joint groups.

	Unadjusted			Adjusted for age, sex, BMI			Adjusted for age, sex, BMI and *OST or ^JSN/DSN of the same joint			Adjusted for age, sex, BMI and radiographic features (OST and JSN) from all joint sites		
Joint Group and Radiographic Feature	β	SE	*p*-Value	β	SE	*p*-Value	β	SE	*p*-Value	β	SE	*p*-Value
**Knee OST**	-0.108	0.028	**<0.0001**	-0.133	0.028	**<0.0001**	-0.151	0.035	**<0.0001^**	-0.110	0.051	**0.0304**
**Hip OST**	-0.038	0.038	0.319	-0.045	0.039	0.241	0.004	0.040	0.911^	-0.029	0.055	0.5963
**Hand OST**	0.0170	0.027	0.527	0.013	0.031	0.667	0.016	0.037	0.663^	0.249	0.050	**<0.0001**
**Spine OST**	0.007	0.030	0.8273	0.006	0.032	0.8625	0.010	0.036	0.7799^	-0.011	0.035	0.7574

**Knee JSN**	-0.043	0.029	0.139	-0.059	0.030	0.051	0.034	0.037	0.335*	0.005	0.050	0.9167
**Hip JSN**	-0.159	0.035	**<0.0001**	-0.165	0.036	**<0.0001**	-0.166	0.037	<0.0001*	-0.207	0.061	**0.0007**
**Hand JSN**	0.008	0.027	0.759	0.004	0.031	0.899	-0.005	0.038	0.884*	-0.261	0.051	**<0.0001**
**Spine DSN**	-0.004	0.031	0.8921	-0.006	0.033	0.8449	-0.011	0.036	0.7689*	0.007	0.036	0.8291

BMI: body mass index; OST: osteophyte; JSN: joint space narrowing; DSN: disc space narrowing; β: parameter estimate; SE: standard error; NS: not significant.

^/* Same joint OST or JSN/DSN variable in models were included after evaluating the models with all joint sites and disease features and backward stepwise selection of the variables

We also used multivariable models that included all joint sites and radiographic features together to identify the predominant site and feature associated with sPIIANP concentrations. Based on ranking by their–log10(p-value), where a value greater than 2 is considered to have a significant effect on the model, hand JSN was the predominant feature associated with sPIIANP, followed by hand OST and hip JSN (Table 4).

**Table 4:** Ranking of the effect of joint and radiographic features in multivariable models.

Joint and Radiographic Feature	-log_10_(*p*-value)
Hand JSN	6.220
Hand OST	6.170
Hip JSN	3.441
Knee OST	1.224
Spine DSN	0.188
Hip OST	0.171
Spine OST	0.134
Knee JSN	0.041

## Discussion

Cartilage homeostasis relies on the controlled catabolism of matrix proteins such as collagen and the replacement with newly synthesized proteins by chondrocytes[2]. We hypothesized that low joint tissue anabolism, as reflected in low sPIIANP concentrations, would be associated with greater radiographic burden of OA at the knee and other joints. Identification of the joint types in which low anabolism (reflected by low PIIANP) is associated with greater OA severity could help target anabolic therapies, currently in development, to joints most in need of such treatments. Our hypothesis was supported by results from knee and hip joints (lower PIIANP associated with greater radiographic burden of disease) but not from spine, which demonstrated no association. These data are consistent with the association of sPIIANP with progression of lower extremity large joint OA[9, 10]. The results for JSN are the most readily interpreted, namely the higher the anabolism (higher PIIANP) the less the hip and knee cartilage loss (reflected by less JSN). The OST results are harder to interpret. In the case of knee OA, the higher the anabolism (PIIANP) the less the OST formation suggesting OST formation may be driven by JSN. Hand OST on the other hand was associated positively with anabolism (higher PIIANP concentration). This could suggest a different pathway of OST formation in knee vs hand joints.

Although analysis of serum PIIANP concentrations in controls was outside the scope of this paper, this important analysis was conducted as part of the Foundation for NIH (FNIH) Osteoarthritis Biomarkers Consortium. In the FNIH study, older multi-joint radiographic controls were assessed for serum PIIANP[21]. These individuals were of comparable age (mean 59 years old) to GOGO and idiopathic OA populations with a median concentration of serum PIIIANP of 2880 ng/ml. More than half of the total knee OA participants in the main FNIH study (n = 600) had the OA risk phenotype, concentrations less than or equal to the median (2880 ng/ml) of the multijoint controls. In our GOGO study, 44.69% of participants had low anabolism (concentrations less than or equal to the median serum PIIANP of the multijoint controls).

Because PIIANP is an anabolic marker, it might be expected that the lack of cartilage collagen synthesis in OA would lead to cartilage loss without a dominant osteophyte component. This is in fact the observed phenotype for hip OA. The dominant finding for the knee, however,was greater OST burden in association with lower sPIIANP. Although significantly greater knee JSN was also observed in association with lower sPIIANP (adjusted for age, sex, and BMI), this was not independent of knee OST. Given the fact that increased COL2A1 gene expression has been identified in early osteophytes[22], these data would suggest that systemic PIIANP does not reflect the local collagen tissue environment in the knee osteophyte but rather a more global cartilage anabolic response.

High familial aggregation (heritability) was previously observed for PIIANP ranging from 57%[23] to 62%[24]. The underlying genetic component however, is yet to be identified. In a cluster analysis, high sPIIANP formed a component with high cartilage oligomeric matrix protein and age that was associated with spine facet OA and disc disease as well as hand OA[24]. It is possible that individuals with low PIIANP have accelerated OA progression in joints under load, such as hips and knees, resulting in a higher burden of large joint OA.

It has been challenging to identify serum biomarkers that relate to osteoarthritis due to the contribution of analyte from many joints. Most studies evaluate one joint to the exclusion of others. We believe a strength of this study was the ability to consider all major joint groups simultaneously to determine what radiographic features and specific joint types contribute most to the serum concentration. With this methodology, this study did in fact identify several joint features/types associated to a greater degree with serum PIIANP concentrations, namely osteoarthritis of knees and hips, even after accounting for spine and hand osteoarthritis, age, sex and body mass index. There were also several limitations of this study. This analysis was based on radiographic evidence of OA that is insensitive to many aspects of disease and does not take into account symptoms. There were limited (single) views of both hands and hips and did not account for facet disease of the spine so the total burden of disease could have been underestimated. This was a cross-sectional study so the association of sPIIANP with OA progression of radiographic features could not be assessed. Although the joints differed by their total radiographic feature scores, we normalized each joint site to the maximum score possible to compare across joints. Finally, we did not account for possible familial relationships that might have increased the homogenetity of results. A future study would be needed to evaluate the generalizability of these results in unrelated individuals and to evaluate the effects of anabolic agents on large joint OA.

## Conclusion

Serum PIIANP, representing collagen synthesis, was lower in individuals with a greater burden of radiographic hip and knee OA. These results are consistent with low anabolic response leading to greater extent of OA in load bearing joints. These results suggest that anabolic reagents might be beneficial for large joint lower extremity OA.
